# Interpulse-Interval-Controlled Nanoparticle Formation in Gas-Phase Burst-Mode Femtosecond Laser Ablation

**DOI:** 10.3390/nano16090519

**Published:** 2026-04-25

**Authors:** Bowen Fan, Tao Lü, Jiang Wang, Guodong Zhang, Zhongyin Zhang, Wei Zhang, Guanghua Cheng

**Affiliations:** 1 School of Artificial Intelligence, Optics and Electronics (iOPEN), Northwestern Polytechnical University, Xi’an 710072, China; fbw0326@mail.nwpu.edu.cn (B.F.);; 2 School of Artificial Intelligence and Automation, China University of Geosciences, Wuhan 430074, China; 3 National Key Laboratory of Science and Technology on Power Beam Processes, AVIC Aeronautical Manufacturing Technology Research Institute, Beijing 100024, China

**Keywords:** burst-mode femtosecond laser ablation, gas-phase nanoparticle generation, interpulse interval, molecular dynamics simulation

## Abstract

The formation and size evolution of gas-phase nanoparticles (NPs) in laser ablation inductively coupled plasma mass spectrometry critically influence aerosol transport, plasma ionization efficiency, and ultimately analytical accuracy. Nevertheless, burst-mode laser ablation, as an efficient and versatile strategy for controlling gas-phase NP size, remains insufficiently explored. Here, we combine experimental investigations and theoretical analysis to elucidate the mechanisms of gas-phase nanoparticle formation and size control by tuning the interpulse interval in burst-mode femtosecond (fs) laser ablation. The mean nanoparticle size exhibits a non-monotonic dependence on interpulse spacing, decreasing with a narrowing size distribution as the interval increases from 0 to 300 ps, and then increasing with distribution broadening at longer delays up to 1000 ps, closely correlating with ablation-crater depth. A characteristic transition at ~300 ps is identified, where both nanoparticle size and crater depth reach a minimum, revealing a critical timescale in pulse–plume–surface interactions. Simulations show that the interpulse interval governs the redistribution of laser energy between the surface and plume, driving a transition from surface-dominated ablation to plume-dominated absorption and partial recovery of surface coupling. This delay-dependent framework provides a unified explanation for nanoparticle formation, where particle size is determined by the competition between plume-mediated fragmentation and surface-driven material supply, and offers a basis for tailoring NP size distributions via temporal pulse shaping.

## 1. Introduction

Nanoparticles (NPs) exhibit markedly size-dependent physicochemical properties [1] and therefore display behaviors in optics [2], electronics [3], and catalysis [4] that are fundamentally distinct from those of bulk materials. These unique attributes endow NPs with substantial potential and research value across a wide range of fields, including optoelectronic devices [5], energy conversion [6], biomedicine [7], and geosciences [8]. A growing body of work has demonstrated that particle size and its distribution are key parameters governing NP performance. They not only directly influence band structure, optical response, reactivity, and interfacial behavior [9,10,11] but are also intimately coupled to nonequilibrium kinetic processes during formation, such as vapor-phase condensation and rapid quenching, and may be accompanied by important geochemical effects, including elemental and isotopic fractionation [12,13,14]. Consequently, achieving precise control over NP size is crucial for elucidating formation mechanisms and advancing multidisciplinary applications. In this context, laser ablation provides a unique platform for bottom-up NP synthesis under highly nonequilibrium yet well-controlled conditions, offering broad material compatibility, high product purity, and tunable processing parameters and has therefore been widely employed for NP fabrication and for probing their formation mechanisms [12,15,16].

Under femtosecond laser irradiation, ultrafast energy deposition initiates a series of nonequilibrium processes, including electronic excitation, electron–lattice coupling, melting, vaporization, and plasma plume formation, followed by rapid cooling and condensation of atoms, ions, and clusters into nanoparticles on nanosecond-to-microsecond timescales [12,16,17]. Previous studies have shown that single-pulse laser parameters such as pulse energy, wavelength, and pulse duration influence ablation dynamics, plume evolution, surface modification and NP size and morphology [18,19,20,21,22,23,24,25]. However, these approaches primarily adjust overall energy density and provide limited control over the temporal structure of energy deposition and intra-plume interactions.

Multi-pulse or pulse-train (burst) laser ablation has emerged as an effective strategy for temporal energy modulation. By introducing multiple sub-pulses with tunable interpulse intervals, burst-mode ablation enables refined control over energy delivery without changing the energy or wavelength of individual sub-pulses. Subsequent sub-pulses can strongly interact with the preformed plasma plume and molten layer, substantially modifying plume dynamics and material ejection behavior, thereby providing additional degrees of freedom for NP nucleation and growth [26,27,28,29,30,31,32,33]. Although extensive studies have investigated burst-mode laser-matter interactions and demonstrated effective NP size control in liquid environments [34,35,36,37], the mechanisms governing NP formation under gas-phase burst-mode laser ablation, where plume expansion and condensation proceed fundamentally differently, remain largely unexplored.

One important application of gas-phase nanoparticles lies in laser ablation inductively coupled plasma mass spectrometry (LA-ICP-MS), where the size, morphology, and size distribution of ablation-generated aerosol particles directly affect aerosol transport efficiency and ionization efficiency in the ICP and, consequently, the accuracy and reproducibility of trace-element analysis [38]. In particular, elemental and isotopic fractionation arising during laser ablation, aerosol transport, and ICP ionization has been recognized as a major limiting factor in LA-ICP-MS, and previous studies have shown that smaller nanoparticles with narrower size distributions can effectively enhance transport and ionization efficiencies while suppressing fractionation effects [39]. Despite this practical importance, the mechanisms governing gas-phase nanoparticle formation under multi-pulse or burst-mode laser irradiation remain poorly understood. In particular, the role of the interpulse interval in regulating energy deposition, plume dynamics, and nanoparticle growth has not been systematically clarified, and direct control of nanoparticle size under gas-phase burst-mode conditions has yet to be demonstrated.

In this work, we combine experiments and molecular dynamics simulations to investigate gas-phase nanoparticle formation under burst-mode femtosecond laser ablation. By tuning the interpulse interval, we demonstrate active control over nanoparticle size and distribution. A pronounced non-monotonic dependence on interpulse delay is identified, with a characteristic transition at ~300 ps, where both nanoparticle size and ablation-crater depth reach a minimum, revealing a critical timescale governing the interaction between successive pulses and the evolving plume–surface system. We further establish a delay-dependent energy deposition mechanism, in which the interpulse interval regulates the redistribution of laser energy between the surface and the plume, driving a transition from surface-dominated ablation to plume-dominated absorption and partial recovery of surface coupling. This framework provides a unified physical picture of nanoparticle formation, where particle size is governed by the competition between plume-mediated fragmentation and surface-driven material supply, supported by the combined experimental and simulation results.

## 2. Materials and Methods

**Figure 1:**
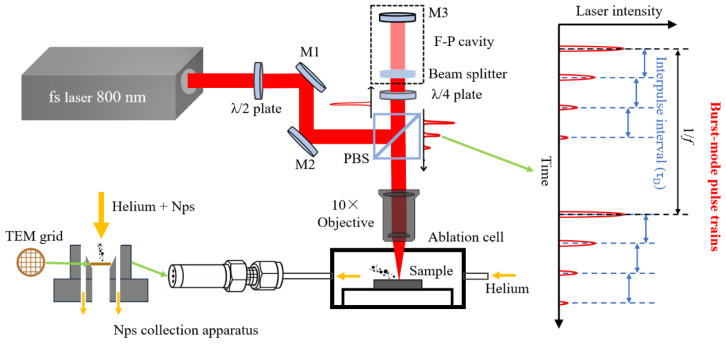
Schematic illustration of the laser pulse-train ablation of the target and the NP collection system.

The laser ablation experimental setup is schematically illustrated in Figure 1, consisting of a three-dimensional (3D) translation stage, beam delivery optics, a pulse-train generation module, a femtosecond laser source, a sealed ablation chamber, and an NP collection unit. A metallic aluminum target was mounted inside a closed ablation cell, into which helium gas was introduced at a controlled flow rate. A Gaussian femtosecond laser beam with a central wavelength of 800 nm was delivered by a commercial Ti: sapphire laser system (maximum repetition rate (*f*): 1 kHz; minimum pulse duration: 35 fs). Unless otherwise noted, the total pulse-train energy was fixed at 100 μJ. The beam was vertically focused onto the target surface using a 10× objective lens (NA = 0.28), corresponding to an incident fluence of 781 J/cm^2^.

Burst-mode pulse trains were generated using a Fabry–Pérot (F–P) cavity configuration. The incident femtosecond laser pulse was partially transmitted and reflected by a beam splitter acting as a partially reflective mirror, while the reflected portion was directed toward a high-reflectivity mirror (M3) and subsequently returned to the beam splitter. Through multiple round trips within the F–P cavity, a sequence of temporally delayed sub-pulses was produced at the output, forming a pulse train. The interpulse interval between adjacent sub-pulses was precisely controlled by adjusting the optical path length of the cavity, that is, the distance between the beam splitter and the high-reflectivity mirror (M3). Varying this distance directly changed the round-trip time of the cavity and thus the temporal spacing between successive sub-pulses. The energy partition among the sub-pulses followed a geometric decay (0.5:0.25:0.125: …), determined by the transmission and reflection coefficients of the beam splitter (50%/50%), with each subsequent sub-pulse carrying half the energy of the preceding one. The overall irradiation time was regulated using a mechanical shutter, while the dwell time at each ablation position was fixed at 2 s using a computer-controlled scanning program. For each nanoparticle collection experiment, a total of 200 ablation spots were processed to ensure sufficient particle yield and statistical reproducibility.

NPs generated during ablation were entrained by the helium (He) carrier gas (0.5 L/min) and deposited onto copper TEM grids. In the present experiments, the He pressure (0.3 MPa) and carrier-gas flow rate (0.5 L/min) were kept constant to eliminate variations in plume confinement and aerosol transport so that the observed nanoparticle-size evolution could be mainly attributed to the variations in the laser parameters. The collected NPs were imaged by transmission electron microscopy (TEM), and their size distributions were determined through statistical analysis.

## 3. Model and Simulation

In the theoretical simulations, a three-dimensional (3D) two-temperature model coupled with molecular dynamics (TTM–MD) was employed to simulate femtosecond laser ablation of metallic aluminum. The governing two-temperature equations are given as follows:

(1) $$C_{e}\frac{\partial T_{e}}{\partial t}=\frac{\partial }{\partial x}\left(k_{e}\frac{\partial T_{e}}{\partial x}\right)-g\left(T_{e}-T_{l}\right)+S\left(x,t\right)$$

(2) $$C_{l}\frac{\partial T_{l}}{\partial t}=\frac{\partial }{\partial x}\left(k_{l}\frac{\partial T_{l}}{\partial x}\right)+G\left(T_{e}-T_{l}\right)$$

where $T_{e}$ and $T_{l}$ are the electron and lattice (ion) temperatures, respectively; $C_{e}$ and $C_{l}$ denote the volumetric heat capacities of the electron and lattice subsystems; $k_{e}$ is the electron thermal conductivity; *g* is the electron–phonon coupling factor that governs the rate of energy exchange between the two subsystems; $S\left(x,t\right)$ represents the laser energy source term describing the spatiotemporal energy deposition; *t* is time; and *x* is the spatial coordinate along the heat-transport direction.

The simulations were performed using the Large-scale Atomic/Molecular Massively Parallel Simulator (LAMMPS) platform [40]. The 3D TTM–MD implementation couples the two-temperature equations with the molecular dynamics model via a coarse-grained electronic temperature grid introduced by Rutherford et al. [41]. To facilitate understanding of the multi-pulse interaction mechanism, only a simplified double-pulse case is considered here, and the laser source term $S\left(x,t\right)$ can be expressed as:

(3) $$S\left(x,t\right)=\left(1-R\right)\frac{1}{\sqrt{2\pi }\sigma_{t}}\left[I_{1}e^{-\frac{{\left(t-t_{1}\right)}^{2}}{2\sigma_{t}^{2}}}+I_{2}e^{-\frac{{\left(t-t_{2}\right)}^{2}}{2\sigma_{t}^{2}}}\right]\alpha_{0}exp\left[-\alpha_{0}\left(x-x_{s}\right)\right]$$

where $S\left(x,t\right)$ is the volumetric laser energy source term; R is the surface reflectivity of the target at the laser wavelength (so 1−R is the absorbed fraction); $\sigma_{t}$ is the temporal standard deviation of the Gaussian pulse (related to the pulse duration); $I_{1}$ and $I_{2}$ are the average intensities (or weighting coefficients proportional to the energies) of the first and second sub-pulses, respectively; $t_{1}$ and $t_{2}$ denote the arrival times (pulse centers) of the two sub-pulses, with $t_{2}-t_{1}$ defining the interpulse interval; $\alpha_{0}$ is the optical absorption coefficient (inverse penetration depth); and $x_{s}$ is the target surface position, such that $\alpha_{0}exp\left[-\alpha_{0}\left(x-x_{s}\right)\right]$ describes exponential attenuation of the absorbed energy with depth into the target.

A 3D atomistic domain of 40.5 × 4.05 × 4.05 nm^3^ containing ~4 × 10^4^ Al atoms was constructed, and interatomic interactions were described using an embedded-atom method (EAM) potential for aluminum. Intervaled double-pulse femtosecond laser ablation of an Al target was simulated. A double-pulse configuration was adopted instead of explicitly modeling a multipulse/pulse-train sequence because it (i) isolates the fundamental role of interpulse interval and reheating in a controlled manner, (ii) captures the essential pulse-plume/pulse-melt coupling mechanisms that also govern later pulses in a burst, and (iii) significantly reduces the computational cost and parameter dimensionality associated with long pulse trains, enabling systematic comparisons across intervals. The model should therefore be regarded as a mechanism-oriented simplification rather than a full quantitative description of the complete burst sequence. The interpulse intervals of double pulses were set to 0, 150, 300, 500 and 800 ps. The total fluence of the double-pulse excitation was 0.48 J/cm^2^, with a sub-pulse energy ratio of 2:1. For aluminum at 800 nm, the complex refractive index was taken as n = 2.7673 and k = 8.3543, yielding an optical absorption depth of 7.62 nm and a reflectivity of 86.82%. During the simulations, the dynamic evolution of the ablation surface was tracked, and the spatial position of the second sub-pulse energy deposition was adjusted according to the evolving crater depth to represent reheating of the transient ablation surface. The electron heat capacity, electron thermal conductivity, and electron-phonon coupling constant of aluminum were adopted from the parameters reported by Roth et al. [42].

One limitation of the present model is that it considers only the absorbed laser energy density and the evolution of the dynamically evolving ablation surface, while neglecting pulse-to-pulse changes in the optical response after the first pulse, such as reduced reflectivity and enhanced absorptivity. By the time the second pulse arrives, the target surface has already developed a structurally complex, foam-like morphology, rendering the magnitude and relevance of these optical-property variations difficult to quantify with confidence. In addition, such geometries with pronounced, multiscale density fluctuations introduce further challenges for atomistic simulations. Owing to the limited simulation domain, structural features such as voids, droplets, and fragments cannot grow to the hundreds-of-nanometers or even micrometer scale, which constrains the direct representation of large-scale surface morphologies.

## 4. Results and Discussion

### 4.1. Experimental Characterization of Nanoparticle Formation and Ablation Behavior

NPs generated by (i) single-pulse laser ablation at different fluences (156.2, 585.8, 781, 976.3, 1366.8, 1952.5, and 2343 J/cm^2^), and (ii) pulse-train laser ablation of an Al target at an incident fluence of 781 J/cm^2^ with interpulse intervals (τ_D_) of 0, 20, 50, 80, 120, 200, 300, 400, 600, and 1000 ps were characterized by TEM. Here, τ_D_ = 0 ps corresponds to the limiting case of burst-mode excitation in which the sub-pulses temporally overlap, whereas single-pulse ablation involves irradiation by an isolated pulse without interpulse interaction; In the burst-mode experiments, the total incident fluence was fixed at 781 J/cm^2^, while the temporal distribution of the deposited energy was varied by adjusting τ_D_.

**Figure 2:**
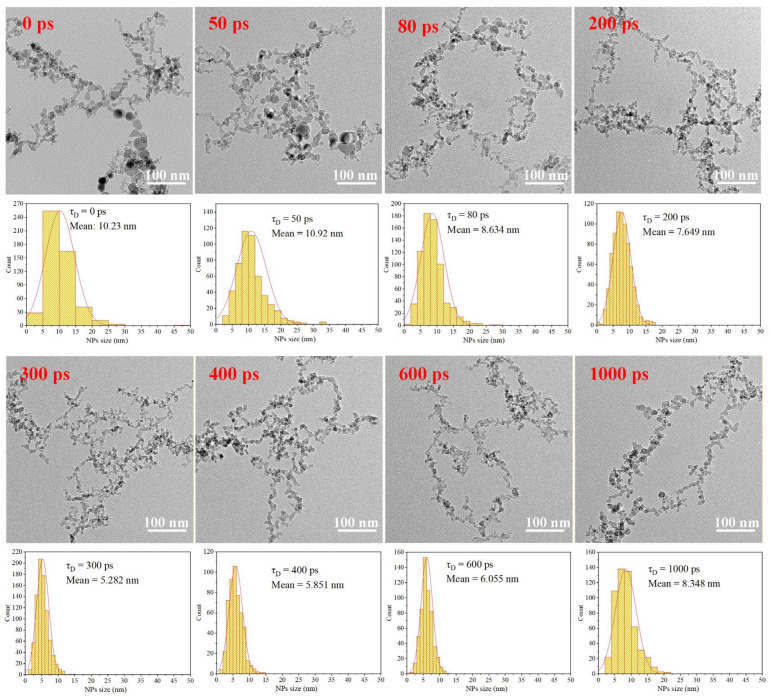
TEM images of NPs produced under pulse-train irradiation with different interpulse intervals (τ_D_), together with the corresponding particle size distribution histograms.

Figure 2 presents representative NP morphologies and the corresponding size-distribution histograms obtained under burst-mode ablation at different interpulse intervals. As τ_D_ increases from 0 to 300 ps, the particle size distribution progressively narrows, indicating a suppression of large-particle formation and the emergence of a more uniform NP population. In contrast, when τ_D_ is further extended from 300 to 1000 ps, the size distribution broadens again, suggesting a recovery of larger-particle contributions.

The filamentous or chain-like nanoparticle structures observed in Figure 2 cannot be explained by a simple uniform particle-collection model, indicating the involvement of post-formation processes. Based on our observations and previous reports (Fieser et al. [43]), these structures are attributed to laser-induced non-thermal surface melting, which creates a transient quasi-liquid state on nanoparticle surfaces. Collisions in this state lead to coalescence at contact points, followed by rapid solidification, resulting in stable chain-like structures. The interparticle connections are inferred to be strong (i.e., solid-state nanojunctions) rather than weak aggregation, as supported by (i) the persistence of chains after washing reported by Fieser et al. [43] and (ii) the presence of necking and fused interfaces in TEM images. Compared with liquid-phase ablation, where rapid quenching suppresses such fusion, the slower cooling in gas-phase conditions prolongs the lifetime of molten or semi-molten surfaces, increasing the probability of particle–particle collisions and enabling effective fusion even at relatively lower pulse energies.

**Figure 3:**
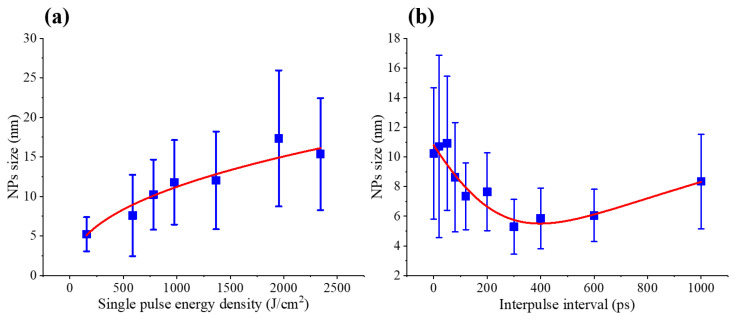
Variations in the mean particle size, together with the corresponding fitting results, for (**a**) single-pulse ablation at different fluences and (**b**) pulse-train ablation at different interpulse intervals.

The corresponding variations in the statistically mean NP size for both single-pulse ablation at different fluences and pulse-train ablation at different interpulse intervals, derived from statistical analysis of the NP size distributions obtained from TEM images (Figure 2), are summarized in Figure 3. As summarized in Figure 3a, under single-pulse irradiation the mean NP diameter increases monotonically with increasing laser fluence, rising from approximately 5.23 nm to 15.37 nm, consistent with enhanced melt ejection and particle coalescence at higher energy densities. By contrast, under burst-mode irradiation, the mean NP size exhibits a pronounced nonlinear dependence on τ_D_, as shown in Figure 3b. Specifically, increasing τ_D_ from 0 to 300 ps leads to a continuous reduction in the mean NP diameter from 10.23 nm to 5.28 nm, whereas further extension of τ_D_ to 1000 ps results in a gradual increase of the mean NP size to approximately 8.35 nm. Together with the evolution of the size-distribution width shown in Figure 2, this behavior indicates that interpulse timing critically modulates the relative contributions of plume-mediated fragmentation and surface-dominated material ejection during NP formation.

**Figure 4:**
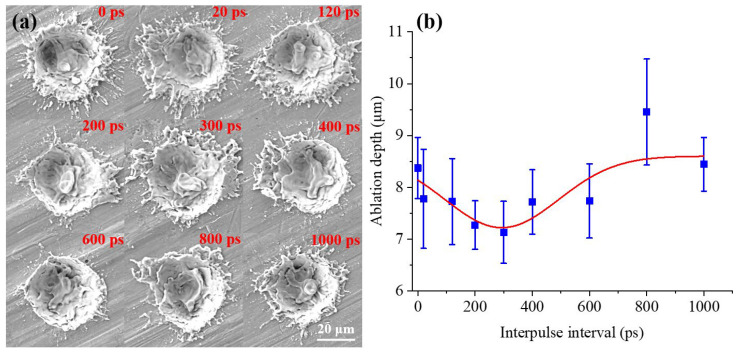
(**a**) SEM images of the ablation craters; (**b**) statistical analysis of crater depths measured by laser confocal microscopy.

To further correlate NP formation with material removal dynamics, pulse trains with different interpulse intervals were applied to ablate multiple locations on the Al target surface using an identical deposited dose of five laser pulses per spot. Representative ablation craters are shown in Figure 4a, and the corresponding crater depths, statistically averaged over 16 craters for each condition, are summarized in Figure 4b. Notably, the dependence of the average crater depth on τ_D_ closely mirrors that of the mean NP size: as τ_D_ increases from 0 to 300 ps, the crater depth decreases from approximately 8.37 μm to 7.13 μm, followed by an increase to approximately 8.44 μm when τ_D_ is further extended to 1000 ps. This correlation suggests a strong coupling between interpulse-dependent energy deposition, material removal efficiency, and NP generation pathways. The mechanistic relationship between ablation-crater evolution and nanoparticle formation will be discussed in detail in Section 4.2 based on molecular dynamics simulations.

### 4.2. Simulation-Assisted Analysis of Plume Dynamics and Nanoparticle Formation

Guided by the experimentally observed dependence of the mean NP size on the interpulse interval, numerical simulations were designed to discriminate the dominant laser–matter interaction mechanisms across different interval regimes. We note, in previous reports [44], that the laser-induced shock wave (SW) represents a compressive stress front propagating into the target and the plume, whereas the subsequent rarefaction wave (RW) induces tensile unloading, promotes plume expansion, and facilitates fragmentation of the ejected material. The competition between these two processes determines whether material removal is dominated by surface ablation or plume-mediated fragmentation, thereby governing nanoparticle formation. However, the present simulations do not permit a rigorous quantitative evaluation of shock- and rarefaction-wave propagation. Because the simulation domain is relatively small, the shock wave propagates beyond the simulation box within the accessible simulation time and is subsequently absorbed by the damping region imposed at the bottom boundary. In addition, the computational cost of large-scale molecular dynamics simulations limits the spatial and temporal scales that can be accessed here. Therefore, the roles of shock and rarefaction waves can only be discussed qualitatively in the present work, based on their inferred effects on plume expansion, recoil-driven backflow, secondary ablation, and fragmentation behavior.

(1) **Short interpulse intervals (** **τ_D_ < 300 ps): surface-coupled regime**

**Figure 5:**
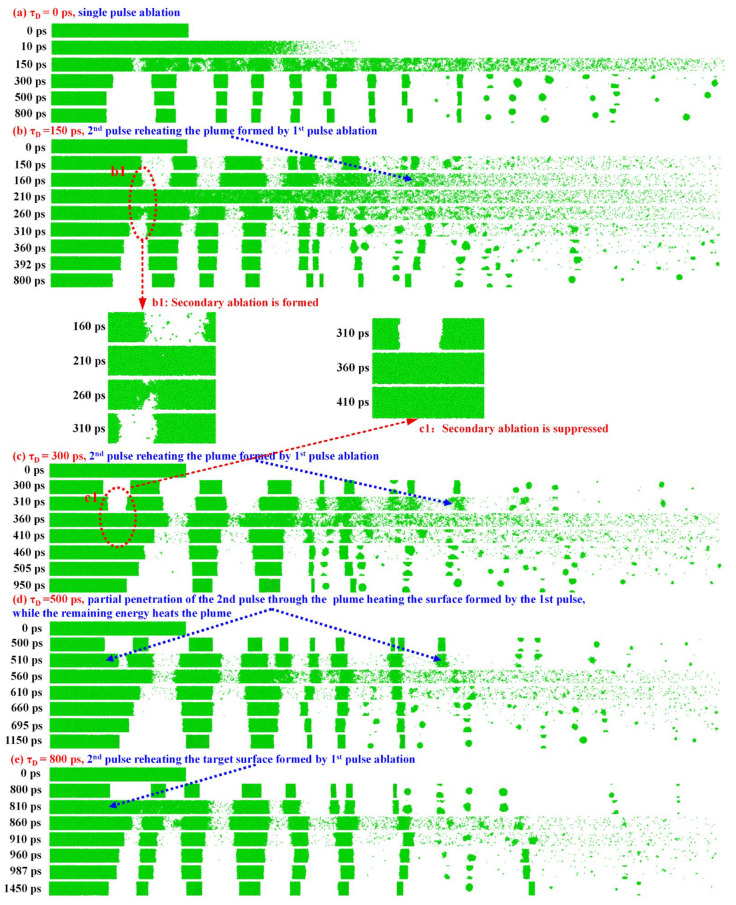
Molecular dynamics snapshots of aluminum ablation under double-pulse irradiation with different interpulse intervals (τ_D_).

As a reference case, Figure 5a corresponds to τ_D_ = 0 ps, representing the limiting case of burst-mode excitation in which the sub-pulses temporally overlap and are equivalent to single-pulse ablation without interpulse interaction.

For an interpulse interval of τ_D_ = 150 ps, as illustrated at position b1 in Figure 5b, the plume reheated by the second sub-pulse remains in close proximity to the target surface and still retains a relatively high density. Under this short-delay condition, the second sub-pulse interacts with a dense near-surface plume, so that part of the deposited energy can be effectively coupled back to the target through recoil-pressure-driven backflow and the return of hot species [29,31]. In addition, the shock wave generated by the second sub-pulse can propagate through the confined plume and further enhance pressure loading at the surface. The subsequent rarefaction wave then extends back to the near-surface region, inducing tensile unloading. The combined action of shock compression and rarefaction-induced unloading promotes the reactivation of secondary ablation, resulting in enhanced material removal and a deeper ablation crater. Meanwhile, the ejected material remains relatively compact and undergoes limited fragmentation, favoring coalescence-dominated growth and ultimately leading to the formation of larger nanoparticles. Experimentally, this regime corresponds to larger nanoparticle sizes (~8.63 nm at 120 ps) and deeper ablation craters (~7.73 μm), indicating efficient material removal.

(2) **Intermediate interpulse intervals (τ_D_~300 ps): plume-dominated regime**

In contrast, when the interpulse interval increases to ~300 ps, as shown in Figure 5c, the plume generated by the first sub-pulse has evolved into a more expanded and optically thicker vapor/plume region. The increased optical thickness leads to stronger absorption and shielding of the second sub-pulse, so that most of its energy is deposited within the plume rather than being directly delivered to the target surface. Although plume reheating can still drive a recoil-induced backflow that transports part of the heated species toward the surface [26,28], the near-surface conditions at this stage are markedly different from those at shorter delays. As indicated at position c1 in Figure 5c, most of the high-temperature material initially accumulated near the surface has already been ejected and expanded away, leaving a significantly cooler and more rarefied near-surface region.

Under these conditions, the shock wave generated by the second sub-pulse is largely confined within the plume, and its coupling to the target surface is substantially weakened. Meanwhile, the rarefaction wave associated with the first sub-pulse continues to propagate, but its tensile driving effect gradually decays with time. Although the plume-confined shock can still induce recoil-driven backflow of ablated species toward the surface, the weakened surface coupling at this intermediate interpulse interval prevents the generation of sufficiently strong tensile unloading to reactivate effective secondary ablation. As a result, material removal is reduced, leading to a shallower ablation crater. In this regime, the decrease in nanoparticle size can therefore be mainly attributed to plume-dominated energy deposition by the second sub-pulse: the limited surface feedback suppresses secondary ablation, while plume expansion and fragmentation become more pronounced, favoring the formation of smaller nanoparticles. Experimentally, this regime corresponds to the minimum nanoparticle size (~5.28 nm) and the shallowest crater depth (~7.13 μm).

(3) **Long interpulse intervals (** **τ_D_ > 300 ps): re-established surface-coupling regime**

As illustrated in Figure 5d, with a further increase in interpulse interval to ~500 ps, the plume has undergone significant expansion and partial dilution, leading to a reduction in its density and optical thickness. Under these conditions, the second sub-pulse can partially penetrate the plume: a fraction of the laser energy is transmitted through the plume and deposited onto the dynamic surface formed by the first pulse, thereby inducing secondary ablation, while the remaining energy is absorbed within the plume and contributes to further plume heating.

As the interpulse interval increases to ~800 ps in Figure 5e, the plume becomes more dilute due to continued volumetric expansion. Consequently, its ability to absorb and shield the incident radiation is further weakened, allowing a larger fraction of the second sub-pulse energy to penetrate through the plume and reach the ablation surface, thereby enhancing direct surface energy deposition. Time-resolved pump–probe measurements by Zhang et al. [45] reported that, at ~1 ns after a 40 J/cm^2^ femtosecond pulse irradiates an Al target, the plume front diameter can reach ~80 μm. On this timescale, the second sub-pulse is therefore expected to penetrate the dilute plume efficiently and deposit most of its energy onto the dynamic ablation surface formed by the first pulse, thereby re-engaging in surface energy deposition and inducing secondary ablation, as depicted in Figure 5e. Under these longer-interval conditions, a new shock wave can be generated near the surface, restoring compressive loading. Consequently, a larger fraction of the second-sub-pulse energy is consumed in renewed surface coupling and secondary ablation, rather than in further fragmentation within the plume, resulting in an increased crater depth and the formation of correspondingly larger nanoparticles. Experimentally, this regime corresponds to an increase in nanoparticle size (~8.35 nm at 1000 ps) and a recovery of crater depth (~8.44 μm).

**Figure 6:**
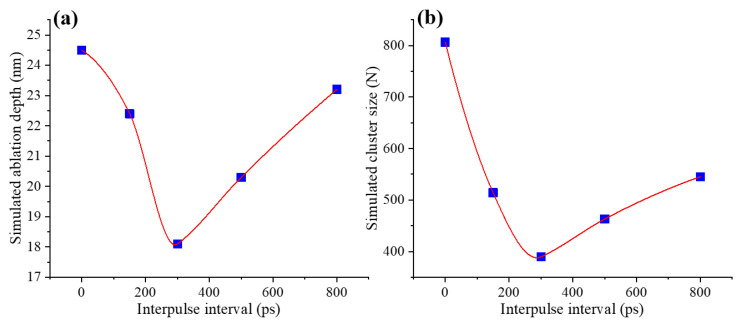
Simulated evolution of (**a**) ablation depth (evaluated at 300 K) and (**b**) cluster size (evaluated at 2000 K) as a function of interpulse interval (τ_D_) in double-pulse laser ablation.

The positions indicated by the black dashed lines in Figure 5a clearly represent the corresponding ablation depth. Upon cooling the system to room temperature (300 K) after femtosecond laser irradiation, the ablation depth was quantified for different interpulse intervals (0, 150, 300, 500, and 800 ps), yielding values of 24.5, 22.4, 18.1, 20.3, and 23.2 nm, respectively, with a minimum at τ_D_ = 300 ps, as shown in Figure 6a. This trend agrees well with the experimentally measured dependence of ablation depth on interpulse interval in Figure 4b.

To further elucidate the mechanisms governing nanoparticle formation under different energy-coupling regimes, we analyze the cluster-size distributions obtained from the simulations. After femtosecond laser irradiation, the system was cooled to 2000 K, and the cluster-size distributions at different interpulse intervals (0, 150, 300, 500, and 800 ps) were evaluated, as shown in Figure 6b. The corresponding mean cluster sizes were determined to be 806.5, 514.1, 389.7, 463.0, and 545.3, respectively, where *N* denotes the number of Al atoms contained in each cluster. Although the accessible cluster-size range is constrained by the finite simulation domain, the results clearly show that at τ_D_ = 300 ps, reheating of the plume by the second pulse enhances fragmentation of the ejected material, resulting in a shift toward smaller clusters after cooling. This trend is consistent with the experimental results shown in Figure 3b, which exhibit a reduction in nanoparticle size at intermediate interpulse intervals. By contrast, when the second sub-pulse couples back to the ablation surface, either through recoil-plume-mediated energy feedback or through partial penetration of the plume, secondary ablation can be reactivated, which tends to increase the size of the ablation-generated nanoparticles.

It should be noted that, the system was cooled to 2000 K, which is above the melting point of aluminum (933 K) but below the vaporization-dominated regime, to represent the high-temperature transient plume environment following femtosecond laser ablation, where nanoparticles are expected to exist in a molten or partially molten state. This choice aims to capture early-stage clustering and aggregation dynamics rather than the final solidified structures. It should be noted that simulations at lower temperatures introduce a bias due to the finite simulation domain, where smaller and faster clusters are more likely to escape through the absorbing boundary, leading to an overestimation of particle size. Therefore, 2000 K represents a compromise between capturing plume dynamics and minimizing boundary-induced statistical bias, while preserving the robustness of the main conclusions.

### 4.3. Future Improvements: Toward Self-Consistent Optical Absorption Modeling

We note, in the present work, that the laser source term is described using a Beer–Lambert-type volumetric deposition with a fixed reflectivity and absorption coefficient. For the second sub-pulse, the deposition position was not determined self-consistently from the evolving optical field, but was prescribed based on the experimentally inferred interaction scenario together with the evolving surface morphology in the simulation. However, it does not explicitly resolve the optical interaction of the subsequent sub-pulse with the inhomogeneous, expanding plume and foam-like near-surface layer generated by the first sub-pulse. In particular, pulse-to-pulse variations in the local dielectric response, reflectivity, absorptivity, and the redistribution of the electromagnetic field within the plume are neglected.

A Helmholtz-based absorption model, as used in previous double-pulse ablation studies, would treat the laser field by solving the wave equation in a spatially varying dielectric medium, with the local absorption determined from [44,46]

$$Q_{L}\left(z,t\right)\propto \omega Im\left[\varepsilon \left(z,t\right)\right]{\left|E\left(z,t\right)\right|}^{2}$$

Under such a framework, the second sub-pulse absorption would no longer be prescribed by a fixed optical penetration depth, but would emerge self-consistently from the evolving density/temperature profile of the plume and transient surface region. Therefore, such a wave-based model would improve the analysis in several important respects. First, it would provide a more realistic description of plume shielding and plume reheating at intermediate interpulse delays. In the current interpretation, the reduction in crater depth and nanoparticle size around 300 ps is attributed to stronger absorption of the subsequent sub-pulse by the expanding plume, which suppresses secondary ablation. A Helmholtz-based model would refine this picture by explicitly resolving where the second sub-pulse deposits its energy within the spatially inhomogeneous plume during the early-to-intermediate stage of laser ablation, and by quantifying the extent to which the plume blocks, redistributes, or feeds energy back toward the surface. In this respect, the PRB 2015 [44] MD–TTM framework is particularly relevant, because its free-electron energy equation explicitly accounts for material motion, allowing a more self-consistent treatment of energy transport and absorption in the evolving plume–surface system.

Second, during the later stage of ablation (300–1000 ps), continued plume expansion is expected to alter the fraction of laser energy absorbed in the plume versus the fraction transmitted through the plume and redeposited at the transient ablation surface. Therefore, in this regime it remains necessary to consider how the laser energy is partitioned between plume absorption and surface absorption, rather than assuming a fixed deposition location or a fixed absorption pathway. A Helmholtz-based treatment would determine this partition self-consistently from the time-dependent dielectric structure of the plume and surface region, thereby providing a more rigorous estimate of when plume-dominated absorption gives way to renewed surface coupling. At the same time, as suggested by Povarnitsyn et al. [44], within the directly simulated delay range the second pulse may still be absorbed predominantly in the plume, whereas renewed direct interaction with the surface becomes increasingly important only as the plume becomes sufficiently dilute and transparent at longer delays.

Therefore, the present analysis should be viewed as a physically motivated interpretation based on a simplified absorption model. Incorporating a Helmholtz-based absorption treatment in future work would mainly strengthen the quantitative description of pulse–plume–surface coupling, especially for the later sub-pulses, by enabling a self-consistent evaluation of the spatial redistribution of laser energy between the plume and the transient material surface.

## 5. Conclusions

This work systematically examines gas-phase nanoparticle formation from an aluminum target under burst-mode femtosecond laser ablation, revealing a nonlinear dependence of nanoparticle size on interpulse interval. The mean nanoparticle size exhibits a nonlinear dependence on interpulse spacing, decreasing from 10.23 nm to 5.28 nm with a narrowed size distribution as the interval increases from 0 to 300 ps and subsequently increasing to 8.35 nm with distribution broadening at 1000 ps. This trend closely correlates with the evolution of ablation-crater depth. Molecular dynamics simulations show that nanoparticle formation in burst-mode femtosecond laser ablation is governed by the delay-dependent interaction between successive sub-pulses and the evolving plume-surface system. The interpulse interval controls a continuous transition from strong plume shielding and surface-coupled energy deposition at short delays, to plume-dominated energy absorption at intermediate delays, and finally to reduced plume shielding with renewed surface coupling at longer delays. The intermediate-delay regime (~300 ps) corresponds to the condition where energy deposition within the plume is maximized, leading to enhanced plume reheating and fragmentation and thus smaller nanoparticles, while material removal is simultaneously reduced. At longer delays, the diminishing plume absorption allows more energy to be deposited at the surface, reactivating secondary ablation and increasing nanoparticle size. A complete physical description of this process requires accounting for both hydrodynamic effects and wave-based absorption mechanisms, which together determine the spatial distribution of deposited energy and the resulting nanoparticle formation pathways.

## Data Availability

The original contributions presented in this study are included in the article. Further inquiries can be directed to the corresponding author.

## Abbreviations

The following abbreviations are used in this manuscript:

NP	Nanoparticle
TEM	Transmission electron microscopy
LA-ICP-MS	Laser ablation inductively coupled plasma mass spectrometry
3D	Three-dimensional
TTM–MD	Two-temperature model coupled with molecular dynamics
LAMMPS	Large-scale Atomic/Molecular Massively Parallel Simulator
EAM	Embedded-atom method potential

## References

[1] Joudeh N., Linke D. (2022). Nanoparticle classification, physicochemical properties, characterization, and applications: A comprehensive review for biologists. J. Nanobiotechnol..

[2] Li H.C., Peng Q.M., Xu X.L., Wang J.P. (2023). Quantum dots for optoelectronics. Adv. Photonics.

[3] De Arquer F.P.G., Talapin D., Klimov V., Arakawa Y., Bayer M., Sargent E.H. (2021). Semiconductor quantum dots: Technological progress and future challenges. Science.

[4] Stewart S., Wei Q.L., Sun Y.G. (2021). Surface chemistry of quantum-sized metal nanoparticles under light illumination. Chem. Sci..

[5] Zhan S.J., Li B.X., Chen T., Tu Y.D., Ji H., Othman D.M., Xiao M.F., Liu R.J., Zhang Z.H., Tang Y. (2025). High responsivity colloidal quantum dots phototransistors for low-dose near-infrared photodetection and image communication. Light-Sci. Appl..

[6] Duan L.P., Hu L., Guan X.W., Lin C.H., Chu D.W., Huang S.J., Liu X.G., Yuan J.Y., Wu T. (2021). Quantum Dots for Photovoltaics: A Tale of Two Materials. Adv. Energy Mater..

[7] Gabriel A.M., Damian-Buda A.I., Brugnari F.M., Camargo E.R., Boccaccini A.R. (2026). Bioactive glass-based core-shell nanoparticles: Multifunctional platforms for controlled drug release and biomedical applications. Mater. Today Bio.

[8] Zhang B.M., Lu Y.X., Wang X.Q., Zhou J., Li H.W. (2025). Metal nanoparticles in soil: Indicators of concealed mineral deposits. J. Geochem. Explor..

[9] Almeida G., van der Poll L., Evers W.H., Szoboszlai E., Vonk S.J.W., Rabouw F.T., Houtepen A.J. (2023). Size-Dependent Optical Properties of InP Colloidal Quantum Dots. Nano Lett..

[10] Andreou E.K., Vamvasakis I., Douloumis A., Kopidakis G., Armatas G.S. (2024). Size Dependent Photocatalytic Activity of Mesoporous ZnIn2S4 Nanocrystal Networks. ACS Catal..

[11] Qin N., Han H., Guan G.J., Han M.Y. (2024). Structurally altered size, composition, shape and interface-dependent optical properties of quantized nanomaterials. Nano Res..

[12] d’Abzac F.X., Beard B.L., Czaja A.D., Konishi H., Schauer J.J., Johnson C.M. (2013). Iron Isotope Composition of Particles Produced by UV-Femtosecond Laser Ablation of Natural Oxides, Sulfides, and Carbonates. Anal. Chem..

[13] Holá M., Ondrácek J., Nováková H., Vojtísek-Lom M., Hadravová R., Kanicky V. (2018). The influence of material properties on highly time resolved particle formation for nanosecond laser ablation. Spectroc. Acta Part B-Atom. Spectr..

[14] Lorazo P., Lewis L.J., Meunier M. (2003). Short-pulse laser ablation of solids: From phase explosion to fragmentation. Phys. Rev. Lett..

[15] Linz N., Freidank S., Liang X.X., Vogel A. (2025). Laser-induced plasma formation and cavitation in water: From nanoeffects to extreme states of matter. Rep. Prog. Phys..

[16] D’Abzac F.X., Seydoux-Guillaume A.M., Chmeleff J., Datas L., Poitrasson F. (2012). In situ characterization of infra red femtosecond laser ablation in geological samples. Part B: The laser induced particles. J. Anal. At. Spectrom..

[17] Perez D., Lewis L.J. (2003). Molecular-dynamics study of ablation of solids under femtosecond laser pulses—Art. no. 184102. Phys. Rev. B.

[18] Noël S., Hermann J., Itina T. (2007). Investigation of nanoparticle generation during femtosecond laser ablation of metals. Appl. Surf. Sci..

[19] Gonzalez J.J., Liu C., Wen S.B., Mao X., Russo R.E. (2007). Glass particles produced by laser ablation for ICP-MS measurements. Talanta.

[20] Yang Y., Lou R., Yuan H.L., Shu Y.Q., Fan W.H., Cheng G.H., Si J.H. (2021). Investigation of the size of nanoparticles formed during femto- and nanosecond laser ablation of zircon. Opt. Eng..

[21] McMahon-puce W., Mu H.R., Juodkazis S., Moss D.J., Chon J.W.M. (2025). Optimal laser fluence for pulsed laser ablation synthesis of silicon nanoparticles in liquid. Opt. Express.

[22] d’Abzac F.X., Czaja A.D., Beard B.L., Schauer J.J., Johnson C.M. (2014). Iron Distribution in Size-Resolved Aerosols Generated by UV-Femtosecond Laser Ablation: Influence of Cell Geometry and Implications for In Situ Isotopic Determination by LA-MC-ICP-MS. Geostand. Geoanal. Res..

[23] Ngo C.V., Liu Y., Li W., Yang J.J., Guo C.L. (2023). Scalable Wettability Modification of Aluminum Surface through Single-Shot Nanosecond Laser Processing. Nanomaterials.

[24] Perrière J., Boulmer-Leborgne C., Benzerga R., Tricot S. (2007). Nanoparticle formation by femtosecond laser ablation. J. Phys. D-Appl. Phys..

[25] Boulmer-Leborgne C., Benzerga R., Perriere J. (2009). Nanoparticle formation by femtosecond laser ablation. Laser-Surface Interactions for New Materials Production: Tailoring Structure and Properties.

[26] Kerse C., Kalaycioglu H., Elahi P., Çetin B., Kesim D.K., Akçaalan Ö., Yavas S., Asik M.D., Öktem B., Hoogland H. (2016). Ablation-cooled material removal with ultrafast bursts of pulses. Nature.

[27] Park M., Gu Y.R., Mao X.L., Grigoropoulos C.P., Zorba V. (2023). Mechanisms of ultrafast GHz burst fs laser ablation. Sci. Adv..

[28] Li X., Jiang L. (2012). Size distribution control of metal nanoparticles using femtosecond laser pulse train: A molecular dynamics simulation. Appl. Phys. A-Mater. Sci. Process..

[29] Fokin V.B., Povarnitsyn M.E., Levashov P.R. (2017). Simulation of ablation and plume dynamics under femtosecond double-pulse laser irradiation of aluminum: Comparison of atomistic and continual approaches. Appl. Surf. Sci..

[30] Obata K., Caballero-Lucas F., Sugioka K. (2021). Material Processing at GHz Burst Mode by Femtosecond Laser Ablation. J. Laser Micro Nanoeng..

[31] Förster G.D., Lewis L.J. (2018). Numerical study of double-pulse laser ablation of Al. Phys. Rev. B.

[32] Wang Q.X., Luo S.Z., Chen Z., Qi H.X., Deng J.N., Hu Z. (2016). Drilling of aluminum and copper films with femtosecond double-pulse laser. Opt. Laser Technol..

[33] Noël S., Hermann J. (2009). Reducing nanoparticles in metal ablation plumes produced by two delayed short laser pulses. Appl. Phys. Lett..

[34] Zhang N., Zhang K.H., Su A.P., Feng C.X., Li J.X., Yang K., Wu D., Hu X.P. Size-controllable synthesis of silver nanoparticles by femtosecond laser pulse train. Proceedings of the 24th National Laser Conference/15th National Conference on Laser Technology and Optoelectronics (LTO).

[35] Li X., Zhang G.M., Jiang L., Shi X.S., Zhang K.H., Rong W.L., Duan J., Lu Y.F. (2015). Production rate enhancement of size-tunable silicon nanoparticles by temporally shaping femtosecond laser pulses in ethanol. Opt. Express.

[36] Yu J.X., Nan J.Y., Zeng H.P. (2017). Size control of nanoparticles by multiple-pulse laser ablation. Appl. Surf. Sci..

[37] Doñate-Buendia C., Spellauge M., Streubel R., Riahi F., Barcikowski S., Huber H.P., Gökce B. (2023). Double-pulse laser ablation in liquids: Nanoparticle bimodality reduction by sub-nanosecond interpulse delay optimization. J. Phys. D-Appl. Phys..

[38] Guillong M., Günther D. (2002). Effect of particle size distribution on ICP-induced elemental fractionation in laser ablation-inductively coupled plasma-mass spectrometry. J. Anal. At. Spectrom..

[39] Zheng X.Y., Beard B.L., Lee S., Reddy T.R., Xu H.F., Johnson C.M. (2017). Contrasting particle size distributions and Fe isotope fractionations during nanosecond and femtosecond laser ablation of Fe minerals: Implications for LA-MC-ICP-MS analysis of stable isotopes. Chem. Geol..

[40] Thompson A.P., Aktulga H.M., Berger R., Bolintineanu D.S., Brown W.M., Crozier P.S., Veld P.J.I., Kohlmeyer A., Moore S.G., Nguyen T.D. (2022). LAMMPS-a flexible simulation tool for particle-based materials modeling at the atomic, meso, and continuum scales. Comput. Phys. Commun..

[41] Rutherford A.M., Duffy D.M. (2007). The effect of electron-ion interactions on radiation damage simulations. J. Phys.-Condes. Matter.

[42] Roth J., Sonntag S., Karlin J., Trichet Paredes C., Sartison M., Krauss A., Trebin H.R. Molecular Dynamics Simulations Studies of Laser Ablation in Metals. Proceedings of the International Symposium on High Power Laser Ablation (HPLA).

[43] Fieser D., Lan Y.C., Gulino A., Compagnini G., Aaron D., Mench M., Bridges D., Shortt H., Liaw P., Hu A.M. (2024). Synthesis and Unique Behaviors of High-Purity HEA Nanoparticles Using Femtosecond Laser Ablation. Nanomaterials.

[44] Povarnitsyn M.E., Fokin V.B., Levashov P.R., Itina T.E. (2015). Molecular dynamics simulation of subpicosecond double-pulse laser ablation of metals. Phys. Rev. B.

[45] Zhang N., Zhu X.N., Yang J.J., Wang X.L., Wang M.W. (2007). Time-resolved shadowgraphs of material ejection in intense femtosecond laser ablation of aluminum. Phys. Rev. Lett..

[46] Povarnitsyn M.E., Itina T.E., Khishchenko K.V., Levashov P.R. (2009). Suppression of Ablation in Femtosecond Double-Pulse Experiments. Phys. Rev. Lett..

